# The Impact of Zinc Oxide Nanoparticles on the Color Stability and Surface Roughness of Heat-Polymerized Maxillofacial Silicone Elastomer Subjected to Artificial Aging: An In Vitro Study

**DOI:** 10.3390/polym17172336

**Published:** 2025-08-28

**Authors:** Lozan Othman, Kawan Othman, Bruska Azhdar

**Affiliations:** 1 Prosthodontic Department, College of Dentistry, University of Sulaimani, Sulaimani 46001, Iraq; 2 Nanotechnology Research Laboratory, Department of Physics, College of Science, University of Sulaimani, Sulaimani 46001, Iraq

**Keywords:** zinc oxide nanoparticles, color stability, surface roughness, maxillofacial silicone

## Abstract

In this study, zinc oxide nanoparticles are utilized to assess the color stability and surface roughness of heat-temperature vulcanized maxillofacial silicone under simulated aging conditions. Silicone specimens were created with different concentrations of ZnO nanoparticles (0 wt%, 1 wt%, 2 wt%, 3 wt%, and 4 wt%) and pigmented with two inherent colors (soft brown and rose silk). The color stability was evaluated by calculating the CIELAB color space, and the surface roughness was analyzed both before and after UV exposure. The applied method considerably improved color stability, with the best results achieved when 1 wt% and 3 wt% ZnO were used. During the aging periods, the soft brown pigment was more resistant to discoloration than rose silk. The incorporation of ZnO resulted in a reduction in the initial surface roughness parameters, while simultaneously increasing the surface’s resistance to UV-induced degradation. Substantial increases in roughness were observed in the control samples. By contrast, adding ZnO improved surface integrity. In conclusion, including an optimized amount of ZnO nanoparticles to heat-polymerized maxillofacial silicone can increase the lifespan of silicone prostheses, providing a smooth appearance and resistance to environmental factors.

## 1. Introduction

Maxillofacial prosthesis is a specialized field of prosthodontics focused on rehabilitating and managing patients with maxillofacial deformities resulting from congenital malformations, trauma, or surgical resection. Extraoral maxillofacial prostheses rehabilitate missing facial structures, enhancing both functionality and appearance, thereby significantly improving patient quality of life [1,2]. Silicone elastomers, commonly known as polydimethylsiloxane, are extensively used in the production of facial prosthetics due to their manipulability, advantageous mechanical and physical properties, and biocompatibility. Their texture closely resembles human skin, providing flexibility and comfort to the patient [3,4,5,6].

Despite these benefits, silicone materials have inherent limitations. A significant issue is the limited clinical lifespan of silicone prostheses, primarily due to color instability and material deterioration. These changes manifest as altered surface roughness and poorly fitting edges, caused by a decrease in tear strength. Color degradation poses a considerable challenge, sometimes necessitating remaking prostheses [7,8]. Successful maxillofacial prostheses heavily depend on accurate color replication, shape, texture, and translucency. These qualities are often achieved through intrinsic or extrinsic pigmentation, with intrinsic pigmentation preferred for its durability, though it is more technically demanding. Previous research has investigated the effect of various intrinsic colors on the mechanical and physical properties of silicone elastomers, aiming to enhance their durability and effectiveness [9]. Various color systems exist to evaluate chromatic differences, including the Munsell color system and the International Commission on Illumination (usually abbreviated CIE for its French name, Commission internationale de l’éclairage) L*a*b* color system. Furthermore, another study cited the use of reflectance spectrophotometry, color analysis, and optical density to assess color stability [8,10]. The mean color stability of an implant-retained auricular silicone prosthesis persisted for 21 months after exposure to water and ultraviolet (UV) radiation [11]. Color alteration in facial silicone prostheses is influenced by several variables, including UV radiation, weathering, the vulcanization process, intrinsic pigment stability, detergents, individual habits, and environmental pollutants [12,13].

Recently, the incorporation of nanoparticles into polymeric matrices has emerged as a promising strategy to improve material properties [2,14]. Nanoparticles enhance the physical and visual attributes of silicone elastomers by forming a three-dimensional network within the polymer chain, thereby improving mechanical strength and UV resistance [15,16,17]. Nanoparticles such as TiO_2_ and ZnO can act as effective shields against UV radiation due to their material properties that enable them to absorb and scatter UV light. Their nanoscale size increases these effects, and they are widely used in sunscreens to block or absorb UV radiation [18]. The main challenge in this endeavor is to maintain an optimal level of nanofiller content while ensuring uniform distribution throughout the formulation. Due to their high surface energy and chemical reactivity, nanoparticles may agglomerate, acting as stress-concentration centers when the silicone elastomer is subjected to external forces, thereby reducing its mechanical strength [17]. The UV-blocking capability of zinc oxide is augmented only by the use of nanosized ultrafine ZnO particles [19,20]. Nanosized ZnO is defined by its diminutive particle size, extensive specific surface area, active functionality, and strong interfacial interaction with organic polymers. Consequently, in addition to augmenting resistance to environmental stress cracking and aging, nanosized ZnO particles also improve the physical and optical qualities of the organic polymer [21,22].

This study aims to assess the impact of zinc oxide (ZnO) nanoparticles, integrated at different concentrations, on the ultraviolet (UV) shielding performance and surface roughness of M511 maxillofacial silicone elastomers during artificial aging. A pigment shade mimicking human skin tone was used to evaluate its resistance to color deterioration, simulating clinical significance, and to enhance the development of durable, realistic face prosthesis, considering both practical and cosmetic factors. The null hypothesis posits that the addition of ZnO nanoparticles neither improves UV protection nor influences the nanoscale surface topography of the material. This investigation introduces for the first time the inclusion of zinc oxide (ZnO) nanoparticles in heat-temperature vulcanized (HTV) maxillofacial silicone elastomers using soft brown and rose silk pigments, which have not been tried before in conjunction with ZnO nanoparticles to assess the color stability of these two pigment shades mimicking human skin tone. Additionally, the surface roughness at the nanoscale of the modified maxillofacial silicone was evaluated, which has not been studied in the previous literature yet.

## 2. Materials and Methods

### 2.1. Materials

The materials were procured from their respective manufacturers, ensuring high quality. These include M511 HTV maxillofacial silicone elastomer (Factor II Inc., Wagon Wheel, AZ, USA) (Parts A and B); nanoparticle ZnO nanopowders with a high purity of 99.95%, a particle size of 18 nm, a specific surface area of 40–70 m^2^/g, a milky white nearly spherical appearance, and a proper density of 5.606 g/cm^3^ (US Research Nanomaterials Inc., Houston, TX, USA); and functional intrinsic skin colors (rose silk and soft brown) sourced from Factor II Inc., USA. The chemicals and reagents were obtained at standard levels and employed without further purification. Absolute ethanol (99.8%, Mw = 46.07 g mol^−1^, from the Merck Company, Rahway, NJ, USA) and ultrapure water (Milli-Q water, with a resistivity of 18.5 MΩ) were used to clean the produced samples. A chromic acid solution and Milli-Q water were used to clean all glassware before use.

### 2.2. Study Design and Sample Preparation

**Figure 1:**
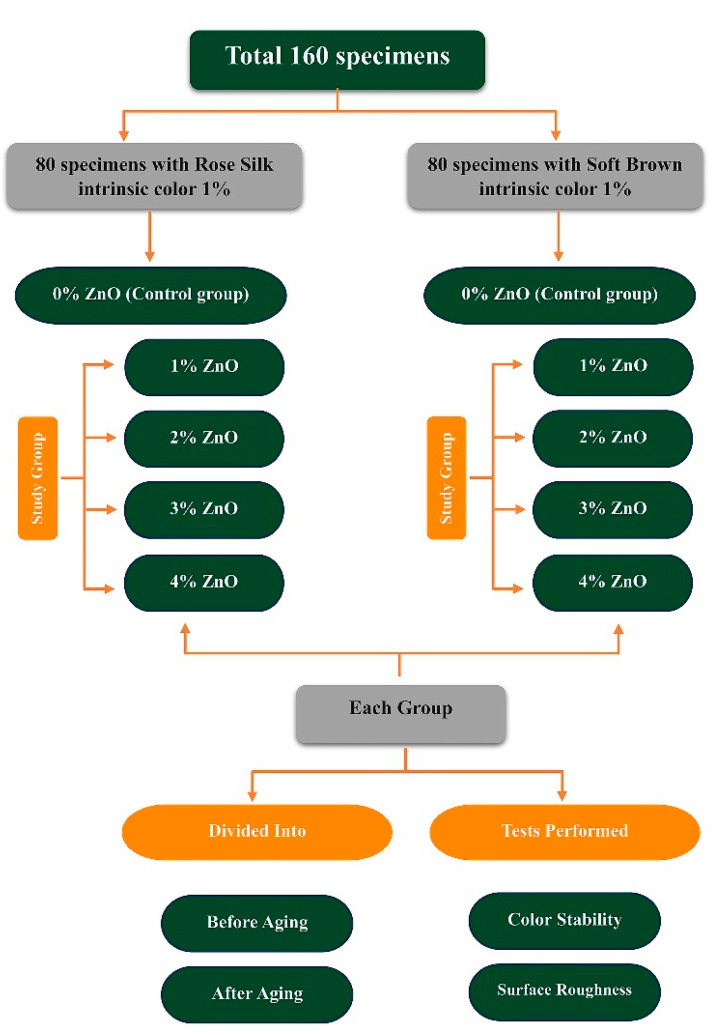
Schematic representation of the distribution of the specimens.

A total of 160 disc-shaped specimens (2 mm thickness; 20 mm diameter) were fabricated utilizing different weight percentages of ZnO nanoparticles (0 wt%, 1 wt%, 2 wt%, 3 wt%, and 4 wt%). The specimens were methodically categorized into 10 groups: 2 control groups, consisting solely of pigment, and 8 experimental groups that incorporated both pigment and ZnO nanoparticles. The two functional intrinsic colors noted above were used, as shown in Figure 1 [9,23,24].

The silicone base and pigment (1%) used for the preparation control group were mixed in a vacuum mixer (AX-2000C; Aixin Medical Equipment Co., Ltd., Tianjin, China) for 10 min at a speed of 360 rpm and a vacuum of −0.09 MPa. After the pigment was added to Part A, the bowl was allowed to cool to reduce any possible impact from residual heat. After cooling, Part B was added, and the mixture was vacuumed for an additional five minutes. As per the manufacturer’s guidelines, silicone parts A and B were combined at a 10:1 ratio [1,2,25,26]. For the study groups, various percentages of ZnO nanoparticles were used (1%, 2%, 3%, and 4%) by weight with Part A and mixed with pigment for 10 min. In the first 2 min, the vacuum was turned off to prevent suctioning the nanoparticles [27].

**Figure 2:**
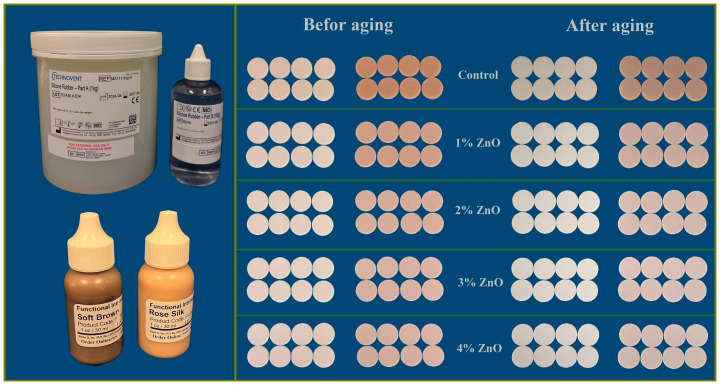
M511 HTV maxillofacial silicone, pigments, and specimens before and after artificial aging.

The filled mold was placed inside a vacuum chamber for 2 min to eliminate any air bubbles produced during pouring. Following this, the mold was placed in a pressure pot at a pressure of 2 bars for two minutes to achieve a smooth surface (PENTOLA A PRESSIONE T1335-00, Lecce, Italy). After a smooth surface was achieved without air bubbles, the molds were closed and subjected to 0.03 MPa of hydraulic pressure for five minutes to maintain the material shape in the mold and remove excess material. G-clamps secured the molds. According to the manufacturer’s instructions, curing was performed in a hot, dry oven at 100 °C for 1 h [28]. The specimens were removed from the molds, rinsed with water and liquid detergent, and dried. All specimens were preserved in a lightproof container to prevent color alterations. Figure 2 illustrates the samples before and after aging with their different concentrations of ZnO NPs, with functional intrinsic skin color and maxillofacial silicone.

Color testing and surface roughness assessments were performed before and after the artificial aging process. Color readings were obtained via a digital colorimeter (WR10QC; FRU, Shenzhen, China). Artificial aging was conducted in a QUV Weather-Ometer chamber according to ASTM G154 Cycle 1, which included UV exposure at an estimated wavelength of 340 nm and an irradiance of 0.89 W/m^2^/nm. Each cycle consisted of 8 h of ultraviolet exposure at 60 ± 3 °C, followed by 4 h of condensation at 50 ± 3 °C [29]. Specimens were evaluated at the beginning and after 252, 504, 1008, 1252, 1504, and 1756 h of aging. The artificial aging condition for 1008 h is equivalent to 1 year of clinical use of the prosthesis, meaning 1756 h is approximately 1 year and 9 months [2].

The color difference (ΔE*) was calculated utilizing the CIE formula [30]:

(1) $$∆E^{*}={\left({∆L}^{2}+∆a^{2}+∆b^{2}\right)}^{1/2}$$

where L, a, and b represent the lightness, red–green, and yellow–blue coordinates, respectively. Surface roughness analysis was performed using Atomic Force Microscopy (AFM; JPK BioAFM, Bruker Optics, Berlin, Germany) [31]. Image processing and color rendering were performed using the Gwyddion 2.66 open-source software [32,33].

### 2.3. Characterizations

X-ray powder diffractometry (XRD) was used to analyze the phase structure of the nanocomposite using a PANalytical X’pert Pro system (Malvern Panalytical, Eindhoven, The Netherlands) with a Cu-Kα radiation source (λ = 0.154 nm, 40 mA, 40 kV) [34]. Atomic force microscopy (AFM) was used to achieve nanometer-scale resolution, providing high-resolution images of surface topography, shape, size, and distribution. Field-emission scanning electron microscopy (FESEM; BestScope Zeiss Sigma VP, Beijing, China) was employed to evaluate the nanoparticle dispersion within the silicone matrix. The chemical bonds and functional groups in silicone were identified using Fourier-transform infrared spectroscopy-attenuated total reflectance (FTIR-ATR; Tensor 27; Bruker Optics, Ettlingen, BadenWürttemberg, Germany). To assess the effects of artificial aging, silicone surfaces were examined before and after the aging process to detect any structural changes [35].

### 2.4. Statistical Analysis

Nonparametric tests were adopted as Shapiro–Wilk tests indicated non-normality. Each ZnO group (1%, 2%, 3%, and 4%) was compared with the control separately at every time point using the Mann–Whitney U test with Bonferroni correction (*p* < 0.01). The Kruskal–Wallis test was used, along with Dunn post–hoc tests (*p* < 0.05), to compare global group differences. Rank–biserial correlation (Mann–Whitney) and epsilon-squared (Kruskal–Wallis) were used to compute the effect sizes. Non-normal data is reported as medians and IQRs. The analysis was conducted using SPSS v28 at a significance level of 0.05.

## 3. Results

### 3.1. X-Ray Diffraction

**Figure 3:**
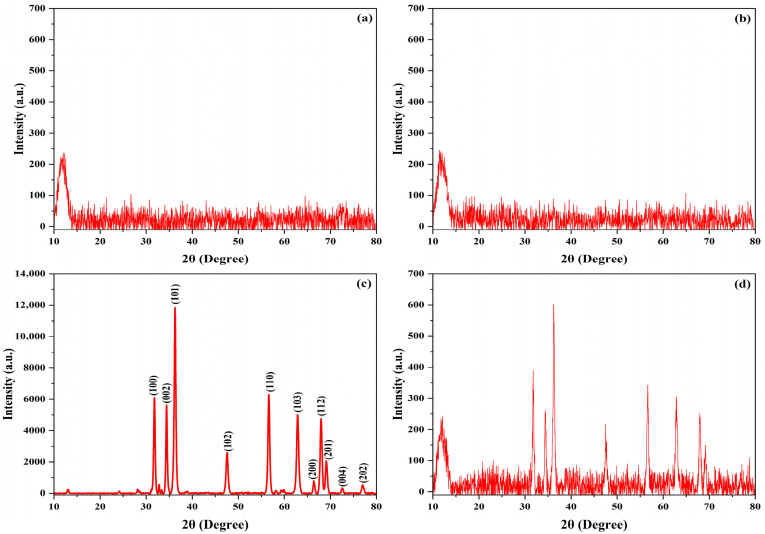
XRD pattern of (**a**) maxillofacial silicon with soft brown pigment, (**b**) maxillofacial silicon with rose silk pigment, (**c**) ZnO nanoparticle powder, and (**d**) silicone–ZnO composite.

The XRD results revealed a distinct peak corresponding to the characteristics of the silicone matrix and the ZnO nanoparticles. The XRD patterns depicted in Figure 3a,b exhibit only a broad, low-intensity peak between 2θ = 10°and 25°, indicating the presence of oriented randomly crosslinked silicon network (-Si-O-Si-) chains [34]. Figure 3c shows high intensity peaks that match the ZnO hexagonal structure with the P 63 m c space group with, reflected peaks at 31.79° (100), 34.43° (002), 36.26° (101), 47.56° (102), 56.63° (110), 62.88° (103), 66.45° (200), 67.98° (112), 69.11° (201), 72.59° (004), and 77.01° (202) [36]. All patterns were identical to ICSD 98-006-7849. Figure 3d shows the superposition of amorphous silicon and ZnO Bragg reflections.

### 3.2. FTIR-ATR

**Figure 4:**
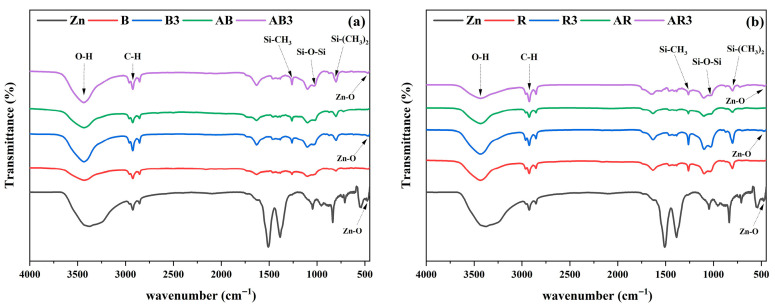
FTIR-ATR spectra of ZnO nanoparticles with maxillofacial silicone blended with (**a**) soft brown and (**b**) rose silk pigments.

Fourier transform infrared spectroscopy-attenuated total reflectance (FTIR-ATR) was employed to detect functional groups and examine structural alterations resulting from the incorporation of ZnO nanoparticles and aging. Figure 4 shows the FTIR-ATR spectra of zinc nanoparticles added to maxillofacial silicone elastomer blended with soft brown (B) and rose silk (R) pigments. Figure 4a illustrates the unmodified zinc alongside the soft brown pigmented silicone (B) and the modified version with 3 wt% zinc before (B3) and after (AB3) aging. Figure 4b presents the rose silk pigmented samples, starting with the control (R), followed by the addition of 3 wt% zinc nanoparticles (R3) before aging. The samples were then subjected to aging (AR and AR3). Figure 4a,b illustrate broad absorption bands ranging from 3144 cm^−1^ to 3662 cm^−1^, which have been attributed to the O-H stretching vibrations of hydroxyl groups resulting from humidity [37]. The intensity of the O-H bands increased with the addition of ZnO nanoparticle fillers, as illustrated in Figure 1. This implies a significant level of water adsorption, which can be attributed to structural defects caused by the nanoparticle bands at 2932 cm^−1^ owing to C-H stretching vibrations [38]. The bands at 470 cm^−1^ show the vibration of the Zn-O nanoparticles [39]. All of the characteristic peaks of maxillofacial silicone were exhibited, including Si-CH_3_ bending vibrations at 1261 cm^−1^, Si-O-Si stretching vibrations at 1030 cm^−1^, and Si-(CH_3_)_2_ bending vibrations at 798 cm^−1^.

### 3.3. FE-SEM

**Figure 5:**
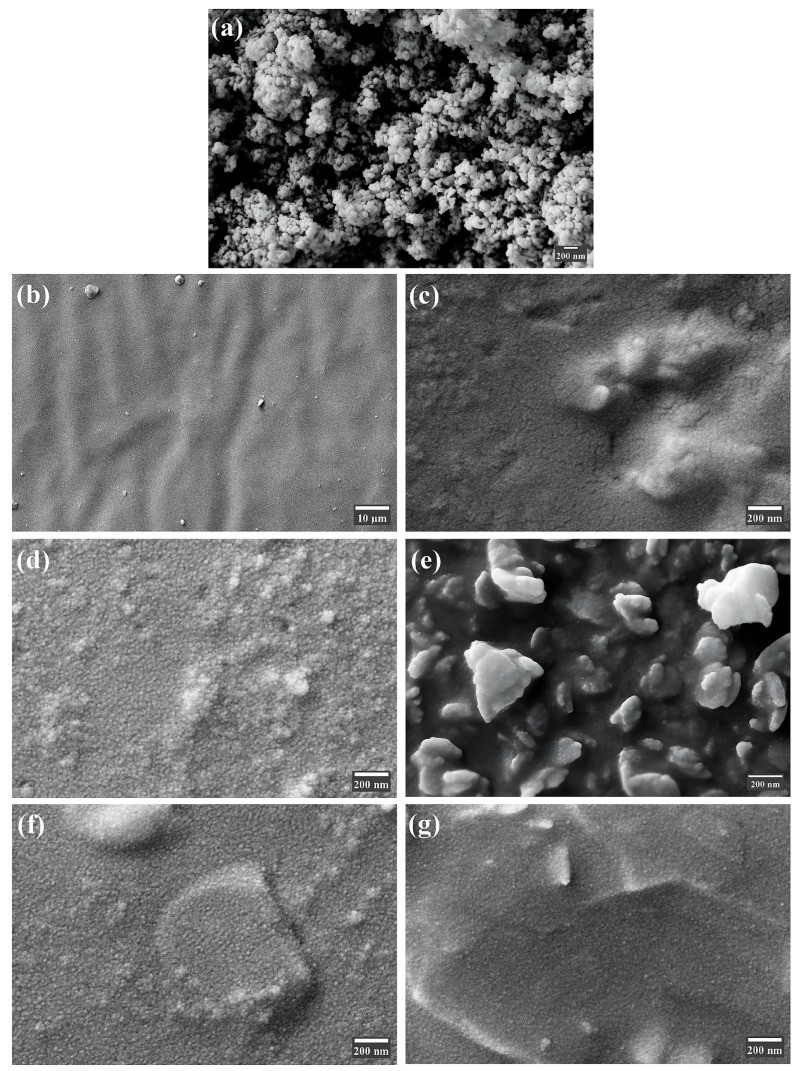
FE-SEM of (**a**) ZnO nanoparticles, (**b**) maxillofacial silicone with soft brown pigment, (**c**) maxillofacial silicone with rose silk pigment, (**d**) soft brown group with 3% ZnO NPs before aging, (**e**) soft brown group with 3% ZnO NPs after aging, (**f**) rose silk group with 3% ZnO NPs before aging, and (**g**) rose silk group with 3% ZnO NPs after aging.

The FE-SEM results confirm that ZnO nanoparticles were incorporated into the maxillofacial silicone matrix, as shown in Figure 5. Figure 5a illustrates ZnO nanoparticle powder that is spherical and highly aggregated due to high surface energy and Van der Waals forces. Figure 5b,c show maxillofacial silicone that is blended with the soft brown and rose silk pigments, without any filler materials present. The surface of the brown pigments appears smoother, with very few surface defects compared with the surface of the soft brown pigments. Figure 5d,e show ZnO nanoparticles blended with maxillofacial silicone at a concentration of 3 wt% for the soft brown pigment, both before and after aging under UV, respectively. Additionally, Figure 5f,g show the rose silk pigment group with 3% ZnO nanoparticles before and after aging. This reveals that ZnO nanoparticles were successfully integrated into the maxillofacial silicone, resulting in morphological changes dependent on the pigment type and aging under UV. The results show that the brown pigment matrix exhibits greater agglomeration after aging, while the rose pigment composite shows better nanoparticle retention and surface stability.

### 3.4. Atomic Force Microscopy

**Figure 6:**
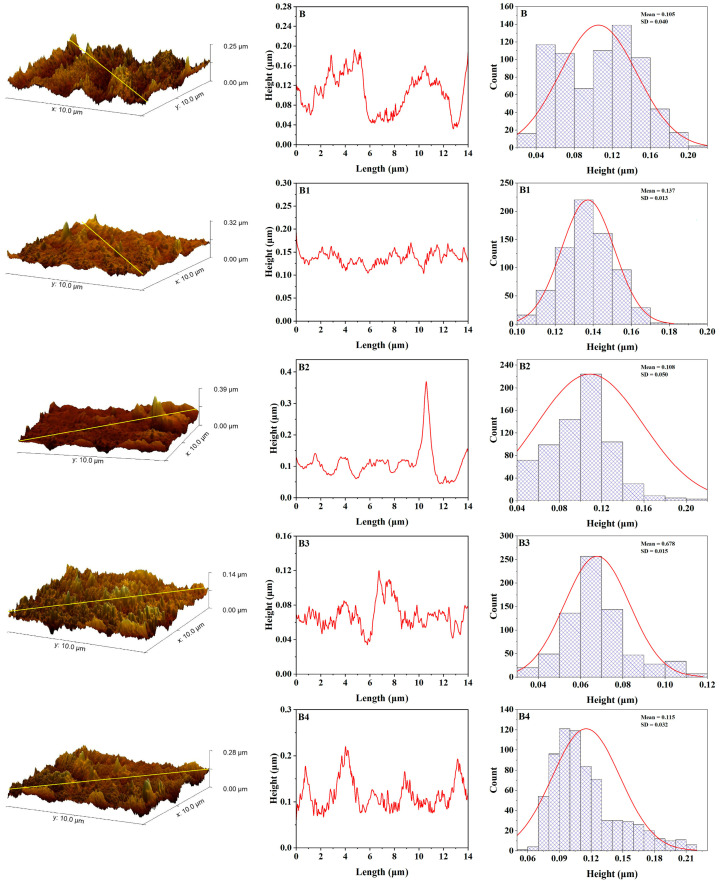
AFM topography, roughness curve, and height distribution for maxillofacial silicone blended with soft brown pigment before artificial aging (B control group, B1 with 1% ZnO Nps, B2 with 2% ZnO NPs, B3 with 3% ZnO NPs, B4 with 4% ZnO NPs).

The surface roughness of maxillofacial silicone elastomers mixed with different amounts of zinc nanoparticles was investigated utilizing Gwyddion for quantitative surface topography analysis. The roughness parameters of maxillofacial silicone elastomers with 3 wt% zinc oxide nanoparticles (R3 and B3 samples) were systematically compared with unmodified control groups (R and B) before and after UV aging. Before aging (Figure 6), control sample B (soft brown pigment) showed an average roughness (Ra) of 5.35 nm; and a root mean square roughness (Rq) of 6.95 nm, while the maximum height of the profile (Rt) was equal to 42.75 nm. However, B3 had lower initial average and peak surface metrics: Ra = 3.53 nm, Rq = 4.55 nm, and Rt = 28.1 nm. These results suggest that zinc nanoparticles have a positive influence on morphology, resulting in a smoother surface. Control sample R in the R series (rose silk pigment) showed values of Ra = 3.45 nm, Rq = 4.39 nm, and Rt = 25.76nm. At the same time, R3 showed moderately higher values, but was still relatively smooth. However, for the R3 sample, the values recorded were Ra = 4.27 nm, Rq = 5.66 nm, and Rt = 49.01 nm, indicating that some nanoparticle dispersion slightly roughens the surface, reducing smoothness due to enhanced morphology from added nanoparticles, as shown in Figure 8.

**Figure 7:**
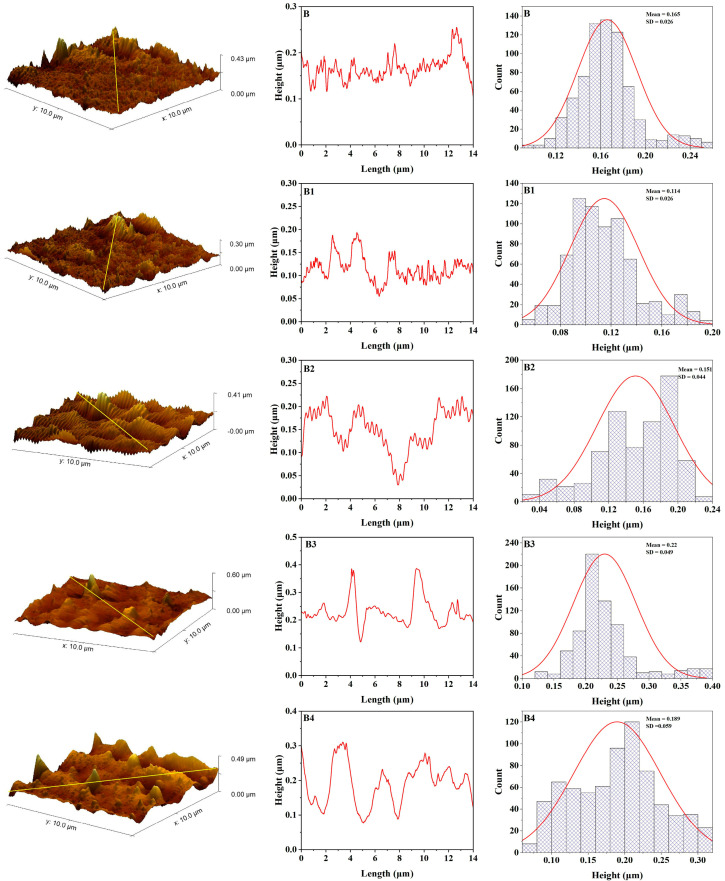
AFM topography, roughness curve, and height distribution for maxillofacial silicone blended with soft brown pigment after artificial aging (B control group, B1 with 1% ZnO Nps, B2 with 2% ZnO NPs, B3 with 3% ZnO NPs, B4 with 4% ZnO NPs).

After UV aging (Figure 7 and Figure 9), both control samples (R and B) exhibited considerable increases in roughness, with B metrics rising to Ra = 7.51 nm, Rq = 9.65 nm, and Rt = 58.38 nm, and R metrics reaching Ra = 3.84 nm, Rq = 5.11 nm, and Rt = 35.15 nm. This alteration signifies surface deterioration caused by chain scission and oxidative mechanisms arising from UV exposure. Conversely, the samples with 3 wt% Zn (B3 and R3) exhibited minimal alterations: B3 aged values were Ra = 4.70 nm, Rq = 6.40 nm, and Rt = 58.93 nm, while R3 attained Ra = 5.34 nm, Rq = 7.43 nm, and Rt = 78.84 nm.

**Figure 8:**
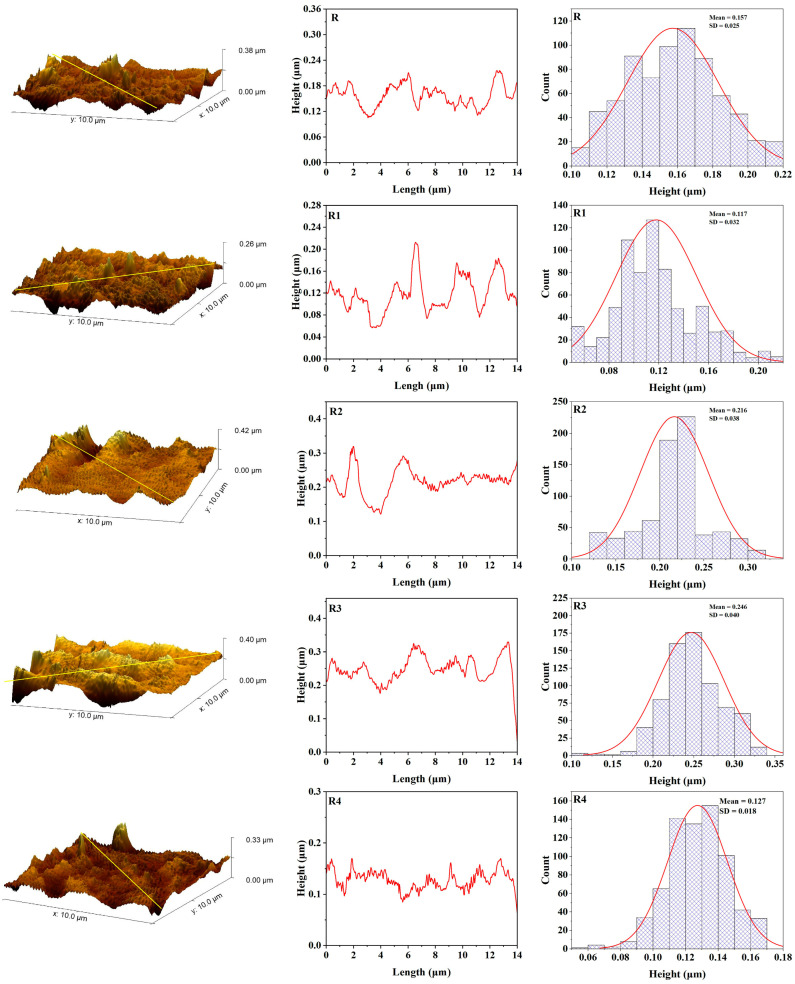
AFM topography, roughness curve, and height distribution for maxillofacial silicone blended with rose silk pigment before artificial aging (R control group, R1 with 1% ZnO Nps, R2 with 2% ZnO NPs, R3 with 3% ZnO NPs, R4 with 4% ZnO NPs).

**Figure 9:**
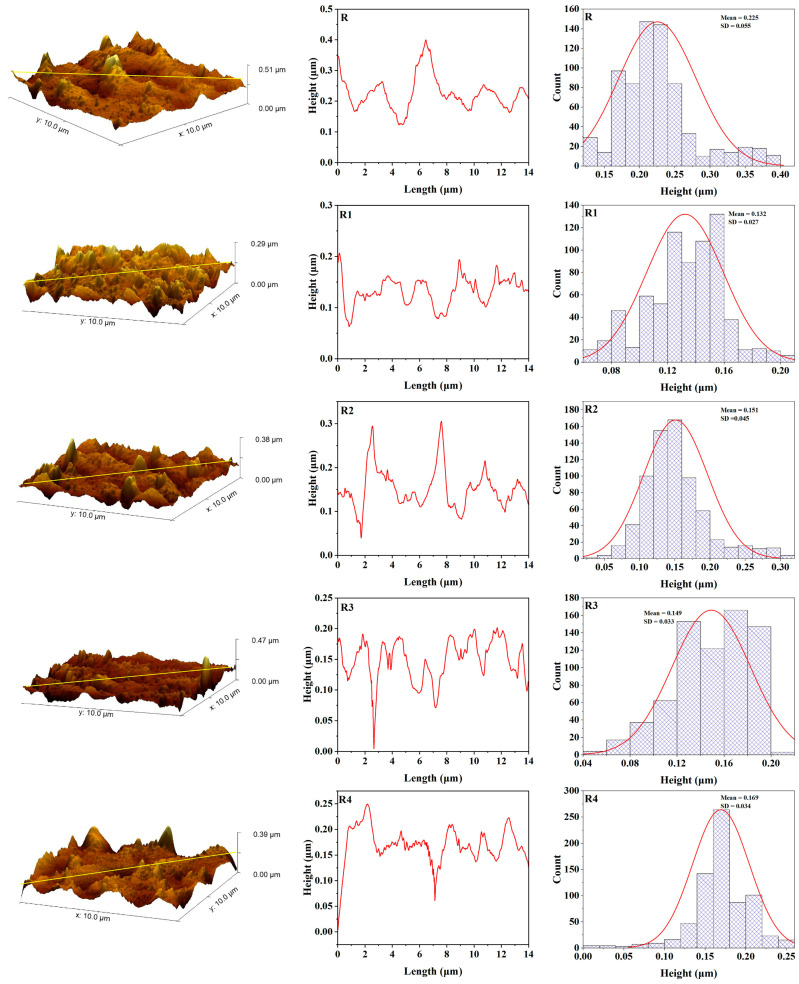
AFM topography, roughness curve, and height distribution for maxillofacial silicone blended with rose silk pigment after artificial aging (R control group, R1 with 1% ZnO Nps, R2 with 2% ZnO NPs, R3 with 3% ZnO NPs, R4 with 4% ZnO NPs).

These findings suggest that while there is a uniform increase in surface roughness from UV aging, zinc nanoparticles, particularly at B3 concentration, lessen surface degradation compared with the control group. The observed protective effect is due to the Zn nanoparticle’s ability to shield UV radiation and its scavenging capability for free radicals, which enhances the photostability of the silicone matrix.

Table 1 shows the average roughness (Ra), root mean square roughness (Rq), maximum height of the profile (Rt) and standard error of samples before and after aging.

### 3.5. Color Stability

The median values of ΔE* and interquartile ranges (Q1–Q3) of both pigments (rose silk and soft brown) in seven acceleration aging periods (252, 504, 756, 1008, 1252, 1504, and 1756 h) of a control and ZnO-enhanced group are presented in Table 2.

For the rose silk pigment control group (0% ZnO), there was a progressive change in median ΔE*, with starting points of 0.13 (0.0836) at 252 h and 0.52 (0.4599) at 1756 h. A significant reduction in the ΔE* value of the ZnO-enhanced samples thus showed more color stability. At 1756 h, the median ΔE* of the ZnO-enhanced groups was as follows: 1%ZnO was 0.24 (0.24–0.27), 2%ZnO was 0.33 (0.20–0.43), 3%ZnO was 0.27 (0.20–0.31), and 4% ZnO was 0.31 (0.24–0.44), and all showed statistically significant improvements compared with the control group (*p* < 0.01).

The same pattern was observed with the soft brown pigment in the control group, which showed an incremental increase in ΔE* as the hours progressed, rising to 0.26 (0.20 to 0.34) after 1756 h, compared with 0.19 (0.13 to 0.25) at 252 h. ZnO nanoparticles significantly decreased ΔE* values at different time intervals. The median ΔE* values of ZnO-containing groups were 0.16 (0.09, 29.32), 0.16 (0.14, 24), 0.19 (0.16, 24), and 0.14 (0.12, 20) at the final time point (1756 h). The 1% ZnO group showed the lowest or nearly the lowest values compared with the others, indicating that it performed best, particularly at earlier time points.

Table 3 shows the results for the rose silk color. Statistically relevant gains were made at 3% ZnO after 504 h, both 1% and 3% ZnO after 756 h, and all ZnO concentrations after the following intervals: 1008, 1252, 1504, and 1756 h. In the case of the soft brown pigment, remarkable deviations were observed at 504 h (1% and 4%), 1008 h (1%, 2%, and 4%), 1252 h (3%), 1504 h (2%, 3%, and 4%), and 1756 h (1%, 2%, and 4%).

The improvements were further validated by effect sizes, which were calculated using the rank—biserial correlation level in the Mann–Whitney U test results and epsilon-squared (2) of the Kruskal–Wallis tests. Statistical analysis was performed using SPSS version 28 with an alpha value of 0.05. These findings indicate the positive effect of ZnO nanoparticles on the development of color stability in well-made maxillofacial silicone materials, as well as the uniformity of the 1% ZnO concentration’s impact on both pigment containers.

## 4. Discussion

### 4.1. Color Stability

The results indicated that adding ZnO nanoparticles significantly reduced ΔE* values over time, especially in the soft brown pigment, which remained more stable than the rose silk at all levels of artificial aging and nanoparticle concentrations. This implies that the color changes in the silicone samples are acceptable for both pigments. The human eye perceives color differences at the perceptibility threshold. By contrast, the acceptability threshold is an acceptable color difference from an aesthetic standpoint [40]. Clinical settings allow materials to change color when the threshold is below the acceptable limit or above the perceivable threshold. This implies that a change in the color of a substance may be detected in the clinic, but it would still appear attractive.

This improved photostability pattern in darker pigments is consistent with Han et al. (2010), who reported that yellow and light-pigmented silicone elastomers had greater ΔE* values than their darker pigmented counterparts under exposure to the artificial aging process [18]. Their results are similar to ours, which showed that rose silk, although it is an improvement over added ZnO, is prone to discoloration compared with soft brown. Relatedly, Abdalqadir et al. (2023) showed that red pigments with or without ZrO_2_ nanoparticles supersede the clinical acceptability limit, ΔE* > 3, whereas mocha pigments retained color stability below human color perception, ΔE* < 1.1 even beyond 1008 h [2]. This evidence substantiates the pigment-based variation in our study, with soft brown (approximated to mocha) emerging as superior to rose silk in all ZnO-enhanced subgroups.

In line with our results, another study by Bangera and Guttal 2014 [22] concluded that ZnO at relatively low concentrations (0.5% and 1.5%) was a more effective and reliable UV blocker than TiO_2_. It was aggravated by a dramatic decline in UV transmission in the case of silicone dispersion with ZnO dopant in silicone [22]. This conclusion is in conjunction with our finding that the 1% ZnO already produced a visible improvement.

An IJISRT report states that ZnO nanoparticles improve the color stability of RTV silicone, resulting in a lower ΔE* value than the control and a better value than TiO_2_ [41]. This finding correlates well with our observations, in which ZnO at 3% had a significant effect on lowering the color changes in the soft brown test samples. Although TiO_2_ outperformed overall, the results demonstrate that ZnO is a functional UV-protective additive, particularly when used in forms optimized for pigment compatibility.

All of these findings support the conclusion that pigment selection, ZnO concentrations, and their interaction in the silicone matrix are essential factors that should be observed to preserve color accuracy in maxillofacial restorations. Despite an overall reduced ΔE* index during the aging period in all instances, soft brown nonetheless displayed better resistance to discoloration than rose silk; however, rose silk also remained within clinically acceptable limits with all the tested concentrations. Therefore, it is possible to claim that both pigments are color-stable, at least when ZnO is optimally supported, which underpins their suitability for use on a long-term basis in clinical practice.

### 4.2. Surface Roughness

The results of this work provide valuable information about the morphology of surfaces and the aging tendency of maxillofacial silicone elastomers, particularly regarding the protective effect of zinc nanoparticle addition against degradation caused by UV exposure. Thorough atomic force microscopy (AFM) measurements conducted in this study provide valuable insights into the parameters of surface roughness and their changes under an accelerated aging regime.

Imaging using AFM quantitative surface topography revealed differences in the morphology of the control and zinc-enhanced samples before and after UV aging. The initial roughness parameters of control sample B (part before aging) were Ra = 5.35 nm, Rq = 6.95 nm, and Rt = 42.75 nm. The addition of 3 wt% zinc nanoparticles into the B3 sample resulted in markedly smoother initial surfaces, at Ra = 3.53 nm, Rq = 4.55, and Rt = 28.1, indicating that nanoparticles have a positive effect on the morphological properties of the silicone matrix.

The increase in surface smoothness resulting from the addition of nanoparticles can be attributed to the interaction between zinc particles and the polymeric silicone network during the curing process. Studies on the dispersion of nanoparticles in polymer matrices have shown that the nucleation sites of well-dispersed nanoparticles can encourage more uniform polymerization and yield improved microstructures [42]. The improvement in surface quality is essential, especially in the context of maxillofacial prosthetics, as smoother surfaces result in less bacterial colonization and are easier to clean, thereby allowing for patient comfort [43].

Significant degradation resistance differences were observed in the accelerated UV aging results compared with the control and zinc-enhanced samples. The control samples revealed substantial changes in surface roughness after exposure to UV, with the B control sample exhibiting substantial changes, as indicated by Ra = 7.51 nm, Rq = 9.65 nm, and Rt = 58.38 nm. Such extensive roughening is consistent with what is known about UV-induced degradation processes in silicone elastomers: chain scission and oxidative degradation ultimately affect the surface of the elastomer [44].

The degradation that occurred in this experiment is consistent with the photooxidative degradation theory discussed in the literature on polymer aging, where UV radiation generates free radicals, which subsequently react with the polymer chain. In particular, silicone elastomers experience molecular chain breakage through the unraveling of silicon—oxygen bonds (Si-O) upon exposure to high-energy UV, decreasing the molecular weight and changing physical properties. The observed surface roughening can be linked to specific surface irregularities and microcracks associated with chain scission events, which contribute to the increase in roughness parameters [45].

The results of this study are adequately grounded in previous research in the field of maxillofacial silicone aging. Alwan et al. found the same pattern with VST-06 silicone elastomers; initially, they become smooth, but with more prolonged weathering, they become rougher [46].

The tear strength and surface roughness assessment conducted by Lanzara proved that even maxillofacial silicone surface properties can be influenced by chemical disinfection procedure [43]. The reported mean surface roughness values of M 511 Technovent (826.26 ± 154.01 nm) were significantly higher than those obtained in the present study using AFM. Therefore, a difference in the determination and surface preparation techniques likely causes this discrepancy. AFM analysis can offer a more precise definition of nanoscale features since it provides an improved level of resolution, surpassing traditional profilometry procedures.

The protective effects observed suggest that zinc-enriched silicone-based products have the potential to extend the service lifetime of prosthetics by maintaining surface integrity upon exposure to the environment. The improvement mentioned above would yield significant advantages for patients, simultaneously diminishing prosthetic replacement rates and costs while preserving the best aesthetic outcome [47].

Pigments significantly impact the surface of maxillofacial silicone materials in various ways, affecting their surface roughness. When integrated with silicone elastomers, pigments provide physical discontinuities within the polymeric network due to the dispersed nature of their particles, which specifically increase texture roughness factors [48]. Various types of pigments exhibit varying impacts on their surfaces. This hypothesis has been confirmed by a recent AFM study, which found that even though no color was observed in samples, dramatic surface roughening occurred in the same samples after artificial aging. By contrast, certain pigments, such as yellow, produced smoother surfaces, while for red, little variation was noted [9]. The pigment dependency of this surface behavior suggests that the chemical composition and particle properties of certain colorants are critical in determining the eventual surface topography and structural durability of maxillofacial prosthetic substances.

## 5. Conclusions

This in vitro study indicates that incorporating zinc oxide nanoparticles (ZnO) throughout the accelerated aging process enhances the photostability and surface resistance of maxillofacial silicone elastomers. The optimal concentrations of ZnO, 1% and 3%, demonstrated exceptional color retention; however, the darker pigment, soft brown, exhibited superior UV stability compared with rose silk. The ΔE* rates were below the threshold of acceptability after 1756 h of aging for both pigments. Atomic force microscopy demonstrated that ZnO enhanced surface smoothness and exhibited resistance to UV-induced roughness compared with the controls, characteristics attributable to the surface integrity and UV protection provided by ZnO. In a clinical context, this would extend the lifespan of prostheses beyond the typical 14 months.

## Figures and Tables

**Table 1:** Average roughness (Ra), root mean square roughness (Rq), maximum height of the profile (Rt), and standard error of samples before and after aging.

Samples	Before Aging				After Aging			
	Ra nm	Rq nm	Rt nm	SE	Ra nm	Rq nm	Rt nm	SE
B	5.35	6.95	42.75	0.00149	7.51	9.65	58.38	0.00097
B1	4.26	5.39	30.9	0.00048	6.84	8.82	64.16	0.00097
B2	4.98	6.99	61.38	0.00186	7.57	8.71	44.1	0.00164
B3	3.53	4.55	28.1	0.00056	4.7	6.4	58.93	0.00182
B4	7.58	9.27	22.53	0.00119	3.36	4.21	26.58	0.0022
R	3.45	4.39	25.76	0.00093	3.84	5.11	35.15	0.00093
R1	3.65	4.66	32.42	0.00119	4.19	5.64	41.01	0.00119
R2	4.3	5.5	39.06	0.00141	5	6.44	54.39	0.00141
R3	4.27	5.66	49.01	0.00149	5.34	7.43	78.84	0.00149
R4	4.72	5.96	35.36	0.00067	3.96	5.33	55.43	0.00067

**Table 2:** Median ΔE* and interquartile range (Q1–Q3) for all groups.

Time (h)	Pigment	Control Median (Q1–Q3)	1% ZnO Median (Q1–Q3)	2% ZnO Median (Q1–Q3)	3% ZnO Median (Q1–Q3)	4% ZnO Median (Q1–Q3)
252	Rose Silk	0.13 (0.08–0.36)	0.14 (0.07–0.25)	0.23 (0.22-0.28)	0.14 (0.11–0.19)	0.17 (0.14–0.25)
	Soft Brown	0.19 (0.13–0.25)	0.13 (0.09–0.21)	0.20 (0.15–0.24)	0.16 (0.12–0.19)	0.12 (0.10–0.21)
504	Rose Silk	0.53 (0.45–0.71)	0.44 (0.42–0.50)	0.54 (0.41–0.59)	0.42 (0.38-0.51) **	0.48 (0.34–0.51)
	Soft Brown	0.45 (0.41–0.52)	0.30 (0.26–0.39) **	0.41 (0.25–0.48)	0.42 (0.38–0.60)	0.34 (0.31–0.37) **
756	Rose Silk	0.29 (0.19–0.47)	0.14 (0.08–0.18) **	0.23 (0.16–0.32)	0.13 (0.07–0.21) **	0.18 (0.13–0.30)
	Soft Brown	0.16 (0.13–0.24)	0.18 (0.13–0.24)	0.18 (0.12–0.24)	0.16 (0.13–0.19)	0.19 (0.17–0.21)
1008	Rose Silk	0.36 (0.26–0.59)	0.16 (0.12–0.18) **	0.24 (0.11–0.40) **	0.20 (0.12–0.26) **	0.21 (0.15–0.28) **
	Soft Brown	0.21 (0.18–0.24)	0.14 (0.10–0.16) **	0.16 (0.13–0.17) **	0.18 (0.13–0.23)	0.14 (0.12–0.24) **
1252	Rose Silk	0.40 (0.34–0.60)	0.18 (0.12–0.20) **	0.15 (0.12–0.21) **	0.16 (0.13–0.23) **	0.20 (0.15–0.23) **
	Soft Brown	0.18 (0.16–0.22)	0.20 (0.16–0.26)	0.18 (0.13–0.25)	0.09 (0.06–0.10) **	0.16 (0.13–0.24)
1504	Rose Silk	0.43 (0.31–0.76)	0.14 (0.07–0.16) **	0.16 (0.09–0.30) **	0.18 (0.10–0.21) **	0.20 (0.14–0.25) **
	Soft Brown	0.21 (0.16–0.25)	0.16 (0.14–0.24)	0.15 (0.12–0.17) **	0.13 (0.10–0.19) **	0.13 (0.12–0.19) **
1756	Rose Silk	0.52 (0.45–0.99)	0.24 (0.24–0.27) **	0.33 (0.20–0.43) **	0.27 (0.20–0.31) **	0.31 (0.24–0.44) **
	Soft Brown	0.26 (0.20–0.34)	0.16 (0.09–0.18) **	0.16 (0.14–0.24) **	0.19 (0.16–0.24)	0.14 (0.12–0.20) **

Key: ** *p* < 0.01 (Mann–Whitney U test vs. control, Bonferroni-corrected).

**Table 3:** Statistically significant ZnO concentration by pigment and time point.

Time (h)	Rose Silk Optimal ZnO% Median (Q1–Q3)	Soft Brown Optimal ZnO% Median (Q1–Q3)
252	0.14 (0.07–0.25)—1% ZnO	0.13 (0.09–0.21)—1% ZnO
504	0.42 (0.38–0.51)—3% ZnO	0.30 (0.26–0.39)—1% ZnO
756	0.13 (0.07–0.21)—3% ZnO	0.16 (0.13–0.19)—3% ZnO
1008	0.16 (0.12–0.18)—1% ZnO	0.14 (0.10–0.16)—1% ZnO
1252	0.18 (0.12–0.20)—1% ZnO	0.09 (0.06–0.10)—3% ZnO
1504	0.14 (0.07–0.16)—1% ZnO	0.13 (0.10–0.19)—3% ZnO
1756	0.24 (0.24–0.27)—1% ZnO	0.14 (0.12–0.20)—4% ZnO

## Data Availability

The data used to support the findings of this study are included within the article.

## Abbreviations

The following abbreviations are used in this manuscript:

Zno	Zinc oxide
NPs	Nanoparticles
HTV	Heat-temperature vulcanized
AFM	Atomic force microscopy
UV	Ultraviolet
CIELAB	Commission internationale de l’Éclairage Lab* color space
ΔE	Color Difference (Delta E*)
XRD	X-ray diffraction
FTIR-ATR	Fourier Transform Infrared Spectroscopy-attenuated total reflectance
FE-SEM	Field-Emission Scanning Electron Microscopy
SPSS	Statistical Package for the Social Sciences
Ra	Average Roughness
Rq	Root Mean Square Roughness
Rt	Maximum Height of the Profile
IQR (Q1–Q3)	Interquartile Range First and Third Quartiles
MPa	Megapascal
nm	Nanometer
